# The role of visual experience in the production of emotional facial expressions by blind people: a review

**DOI:** 10.3758/s13423-017-1338-0

**Published:** 2017-06-23

**Authors:** Dannyelle Valente, Anne Theurel, Edouard Gentaz

**Affiliations:** 1 SensoriMotor, Affective and Social Development Laboratory, Faculty of Psychology and Educational Sciences, 40 bd du Pont d’Arve, 1205 Geneva, Switzerland; 2 The National Center of Competence in Research “Affective Sciences – Emotions in Individual Behaviour and Social Processes” (NCCR Affective Sciences), University of Geneva, 40 bd du Pont d’Arve, 1205 Geneva, Switzerland

**Keywords:** Emotion, Facial Expressions, Blind individuals, Visual experience, Non-visual processes

## Abstract

Facial expressions of emotion are nonverbal behaviors that allow us to interact efficiently in social life and respond to events affecting our welfare. This article reviews 21 studies, published between 1932 and 2015, examining the production of facial expressions of emotion by blind people. It particularly discusses the impact of visual experience on the development of this behavior from birth to adulthood. After a discussion of three methodological considerations, the review of studies reveals that blind subjects demonstrate differing capacities for producing spontaneous expressions and voluntarily posed expressions. Seventeen studies provided evidence that blind and sighted spontaneously produce the same pattern of facial expressions, even if some variations can be found, reflecting facial and body movements specific to blindness or differences in intensity and control of emotions in some specific contexts. This suggests that lack of visual experience seems to not have a major impact when this behavior is generated spontaneously in real emotional contexts. In contrast, eight studies examining voluntary expressions indicate that blind individuals have difficulty posing emotional expressions. The opportunity for prior visual observation seems to affect performance in this case. Finally, we discuss three new directions for research to provide additional and strong evidence for the debate regarding the innate or the culture-constant learning character of the production of emotional facial expressions by blind individuals: the link between perception and production of facial expressions, the impact of display rules in the absence of vision, and the role of other channels in expression of emotions in the context of blindness.

## Introduction

From birth to adulthood, emotions, whether expressed or perceived as expressed by others, remain essential common references for effective social interaction (Matsumoto & Willingham, 2009; Sander & Scherer, 2009). While internal subjective states may not be observed, they are often expressed or communicated through a range of channels including nonverbal vocalizations (Sauter, Eisner, Ekman, & Scott, 2010), posture (Aviezer & Todorov, 2012; de Gelder, 2006), prosody (Adolphs, Damasio, & Tranel, 2002; Frick, 1985), chemosensory signals (Mujica-Parodi et al. 2009), music (Sievers, Polansky, Casey, & Wheatley, 2013) and language (Rimé, 2005), but are conveyed mainly through facial expressions (Ekman, 1992, 1993).

The facial musculature is capable of over 40 independent actions, resulting in an extremely large number of possible expressions. But of this large potential repertoire, strong evidence now exists that a small number of specific facial configurations are universally and discretely produced when emotions are elicited (Ekman, 1993). Indeed, Ekman and his colleagues (Ekman, 1992; Ekman & Friesen, 1971; Ekman, Friesen, & Ancoli, 1980; Ekman, Levenson, & Friesen, 1983; Ekman & Oster, 1979) posited the existence of a limited number of basic, pure emotions that are constrained by physiology, and provoke specific responses in the facial musculature and autonomic system: joy, sadness, fear and anger, possibly supplemented by disgust, surprise, interest or contempt (for a discussion, see Gendron & Barrett, 2009).

One theoretical position suggests that universal expressions originate mainly from an evolved emotion-response system and are a product of our evolutionary history (Darwin, 1972). This position suggests that facial configurations are genetically coded for all humans and are part of a larger response system involving cognitive, physiological and phenomenological processes. According to this view, this coordinated response system is produced from a biologically resident source that requires little or no learning. In the 1960s, evidence of this position was principally reinforced by cross-cultural studies in the recognition of emotions performed by Ekman and colleagues. Across members of different cultures, a high level of agreement was found in the interpretation of facial expressions in photographs of basic emotions (Ekman, 1993; Ekman & Friesen, 1971; Ekman & Oster, 1979). According to Ekman, the effect of learning is minor and consists of modelling these innate expressions. The same configurational pattern is present even if some inter-individual variations can be found in compliance with socially shared expressive codes and display rules reflecting, for instance, differences in intensity of the emotion in a specific context and culture-specific prescriptions regarding who can show which emotions, to whom and when (Ekman, 1993).

However, a second position suggests that universal expressions are produced by culture-constant learning (Mead & Gordan, 1976). In this view, individuals around the world learn, through observational learning, modeling and reinforcement, to associate the same facial configurations with the same emotional states or antecedent events. Facial expressions of emotion, thus, are universal because the same expressions are observed and modeled around the world in response to the same types of emotionally evocative situations. This view is supported by the social and dynamic theories of emotion and its development (Fogel et al. 1992). For the supporters of these theories, the role of learning is central because emotional programming begins and is developed through visual interaction between the baby and his entourage (for a discussion about theories of emotional development, see Galati, Miceli, & Sini, 2001).

This article surveys 21 studies on the production of emotional facial expressions by blind people from birth to adulthood. These studies are doubly interesting from a theoretical point of view. First, they allow the test of some opposing theories concerning the origin and development of the ability to produce emotional facial expressions. Does this ability emerge only after a visual experience during which infants learn to reproduce with their face the emotional facial expressions perceived in their environment, or is it present at the start of life and dependent on inherent structures of the systems involved? Indeed, because blind individuals cannot, from birth or shortly thereafter, see others’ expressions, they cannot learn to produce expressions by modeling. Thus, if congenitally blind individuals express facial emotions in the same way as sighted individuals, this would be compelling evidence that this behavior does not involve visual learning.

Second, the surveyed studies might also answer the question of the role of other non-visual processes, like vocalizations and tactile cues, in the context of blindness, and whether these other perceptual sources play a role in the context of blindness, not only in producing facial expressions but also in discriminating the facial expressions of others. Henceforth, non-visual explanations must be found to account for the existence of the same production of emotional facial expressions in both populations, as different causes can have the same effects. To discover these explanations, we need to know if the factors that influence the production of the facial expressions by blind people are identical to those affecting this behavior in sighted people.

In order to examine these questions, we searched for studies that met the following inclusion criteria: (1) studies concerning the production of facial expression of basic emotions by blind people of all ages, (2) scientific papers published in an international journal, and (3) use of observational or experimental approaches. A search of scientific databases (e.g., Google Scholar, PsycArticles, PsycINFO, ScienceDirect) with the keywords “emotional + blind”, “emotion + blind”, “facial expressions + blind”, “expressive + blind” yielded 20 articles that met all three of our inclusion criteria. Considering there were very few studies available about the production of facial expressions by blind subjects, we also included one article (Kunz, Faltermeier, & Lautenbacher, 2012) analysing facial expressions of pain, even though this is not considered a basic emotion according to the Ekman model (Ekman, 1993).

In particular, we identified and reviewed eight older studies with an observational approach (Dumas, 1932; Eibl-Eibesfeldt, 1973; Fraiberg, 1975; Freedman, 1964; Fulcher, 1942; Goodenough, 1932; Thompson, 1941; Webb, 1977) and 13 recent studies with an experimental approach (Chiesa, Galati, & Schmidt, 2015; Cole, Jenkins, & Shott, 1989; Galati et al. 2001; Galati, Scherer, & Ricci-Bitti, 1997; Galati, Sini, Schmidt, & Tinti, 2003; Kunz et al. 2012; Matsumoto & Willingham, 2009; Ortega, Iglesias, Fernandez, & Corraliza, 1983; Peleg et al. 2006; Rinn, 1991; Roch-Levecq, 2006; Tracy & Matsumoto, 2008; Tröster & Brambring, 1992).

After a discussion of three methodological considerations, this review of studies reveals differing capacities in blind subjects to produce spontaneous or voluntarily posed expressions of emotion. Results provided evidence that visual experience is not necessary to spontaneously produce adequate facial expressions for basic emotions such as happiness, anger and fear. In contrast, results from studies in which subjects were invited to simulate an emotion in a laboratory condition suggest that visual experience affects the production of voluntary expressions and their control.

### Methodological considerations

Three methodological considerations must be taken into account in the study of the production of emotional facial expressions by blind people. The first concerns the comparison of different populations with different visual experiences: sighted subjects, late blind subjects and congenitally blind subjects (Hatwell, Streri, & Gentaz, 2003; Heller & Gentaz, 2014). In fact, the population of people who are blind is characterized by wide interpersonal variability due to the type of visual pathology and age of diagnosis. There are a small number of people who are completely blind since birth, and some have pathologies entailing additional handicaps. This interpersonal variability explains the well-known difficulty researchers confront in assembling an acceptable sample of participants with the same profile of visual impairment. Some of the oldest studies have investigated only small samples of participants, sometimes only a single case. These difficulties in constituting equivalent groups may account for some contradictory results found in the literature.

The second problem concerns the measures used to evaluate the adequacy of emotional expression of blind participants and sighted individuals. In the studies of several pioneers, analyses depended on only subjective interpretations of the researchers or assistants (Eibl-Eibesfeldt, 1973; Fraiberg, 1975; Freedman, 1964; Goodenough, 1932; Thompson, 1941) so it is sometimes difficult to know exactly how these expressions occurred in the blind subjects. To avoid this problem, some studies used visual judgment tasks, in which the adequacy of facial expressions produced by blind individuals is determined by the rate of recognition by naive sighted judges. But, as was pointed out by some researchers (Galati et al. 2001; Galati, Scherer, & Ricci-Bitti, 1997; Ortega et al. 1983), the results stemming from these judgment tasks must also be interpreted with caution. Indeed, it is rather common that the facial expressions of the blind participants are considered inadequate by sighted judges, who may be misled by specific characteristic body and head movements noted in blind individuals, such as eye pressing, body rocking, head or eye rotations, and eyebrow raises. These stereotypic and repetitive behaviors are known in the literature as “blindisms” (Brambring & Tröster, 1992; Chiesa et al. 2015; Leonhardt, 1990). Some have a functional purpose and are used to obtain information from the surroundings. (For instance, totally blind subjects can make head movements to maximise auditory information; people with remaining light perception can engage in repeated eye pressing to obtain perceptual sensations.). Recently, Alfaro (2014) presented and discussed these behaviors which some authors label, sometimes too quickly, “autistic-like”. In any case, they do not have a communicative meaning or a connection with an emotional state. Recently, Chiesa, Galati and Schmidt (2015) analyzed in more detail the impact of these behaviors on interactions between visually impaired mothers and their sighted children. Authors highlighted behaviors that can sometimes confuse sighted interlocutors, for instance, lowering the brow while smiling (Chiesa et al. 2015).

As we will see, several studies used videotapes in judgement tasks comparing facial expressions of blind and sighted individuals (see Table 1 and Table 2 for stimuli used in each study). In these studies, the eventual presence of atypical movements of the body and head, at times contradictory with some expressions, might partially explain some of the poor performance of subjects in these judgment tasks. These movements can also be captured in photographs, producing some atypical static facial features, such as raised eyebrows, open mouth, closed eyes, or unfocused gaze. This question was pointed out by Galati et al. (1997) in his emotion recognition study based on photographs of facial expressions elicited in blind adults.

**Table 1:**
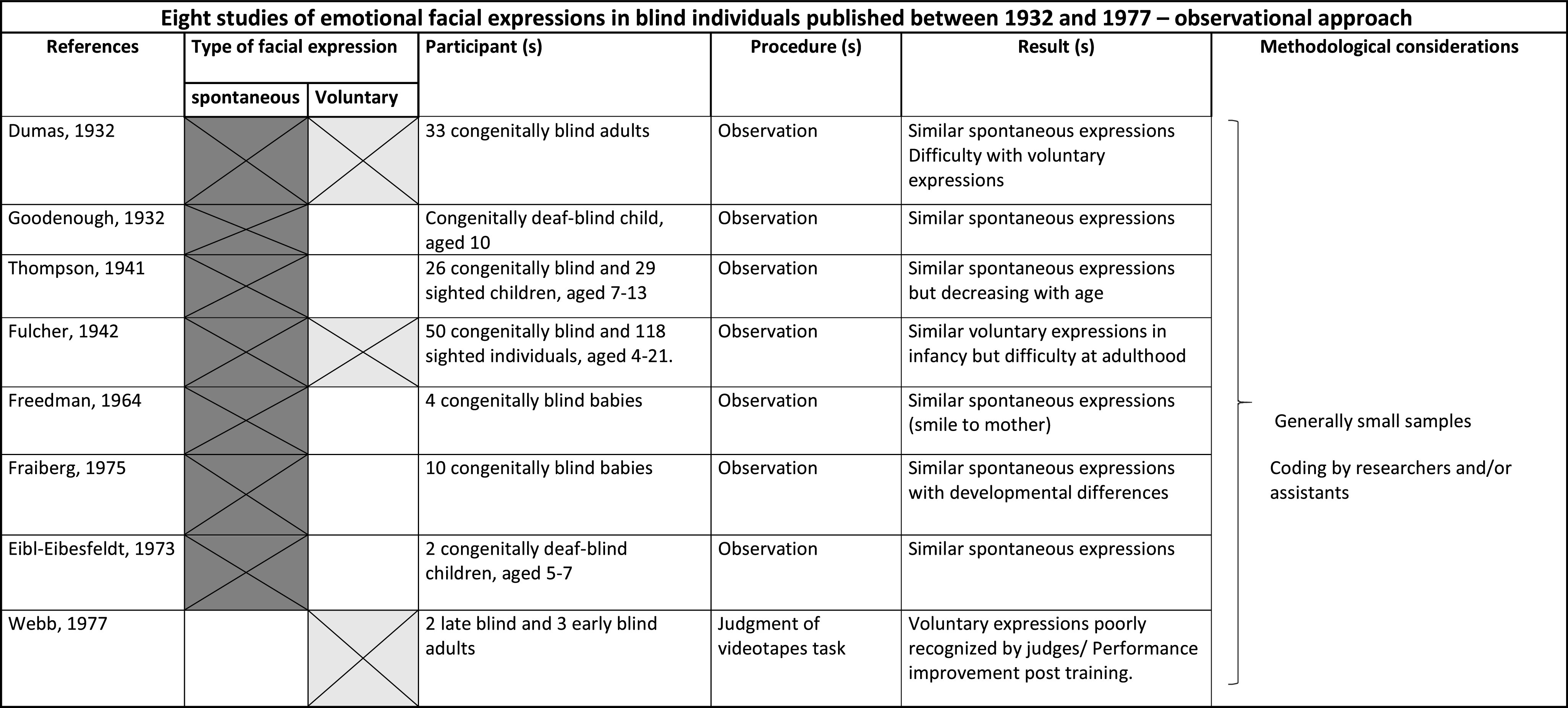
Studies of emotional facial expressions in blind individuals—observational approach

**Table 2:**
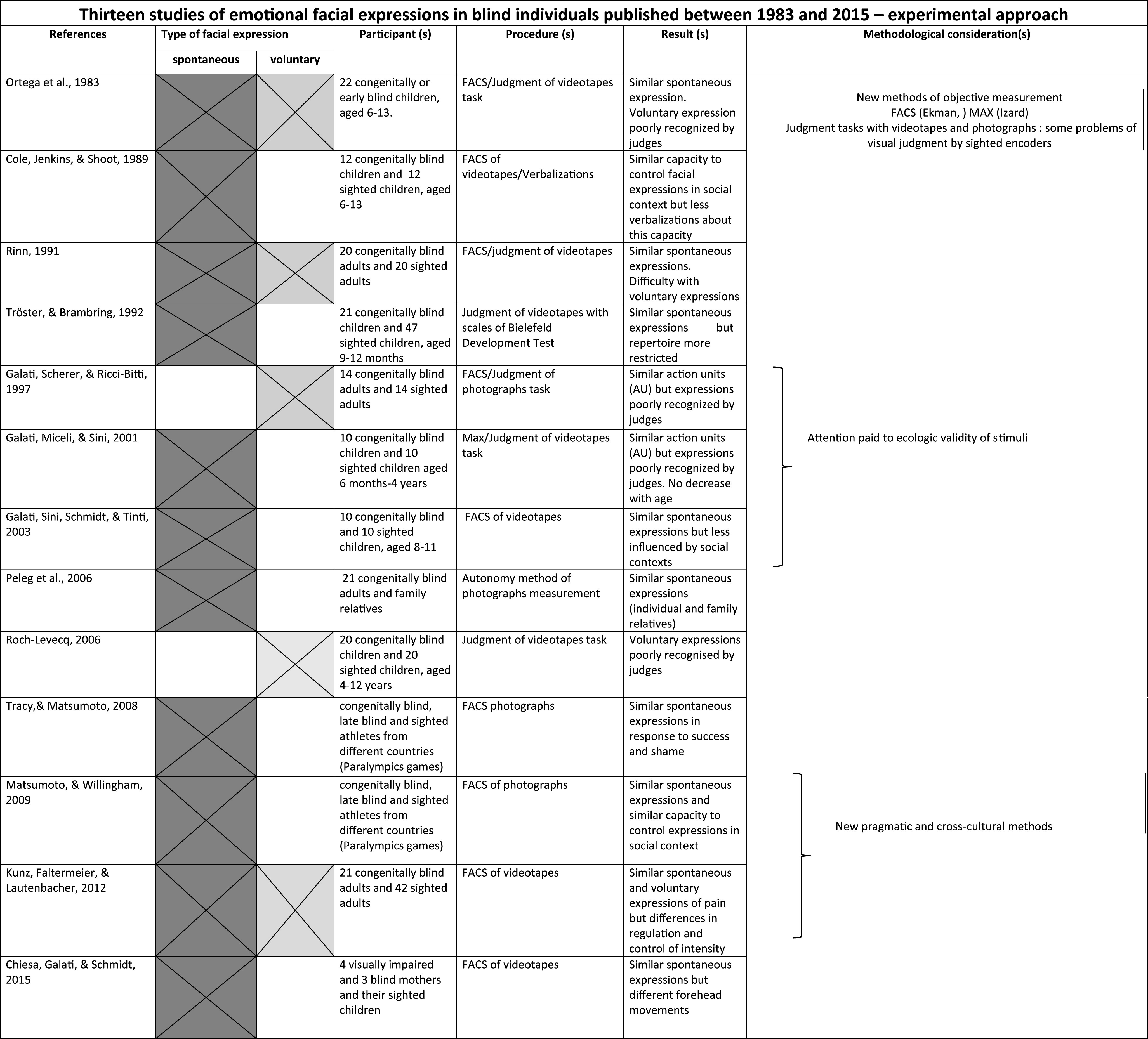
Studies of emotional facial expressions in blind individuals—experimental approach

**Fig. 1:**
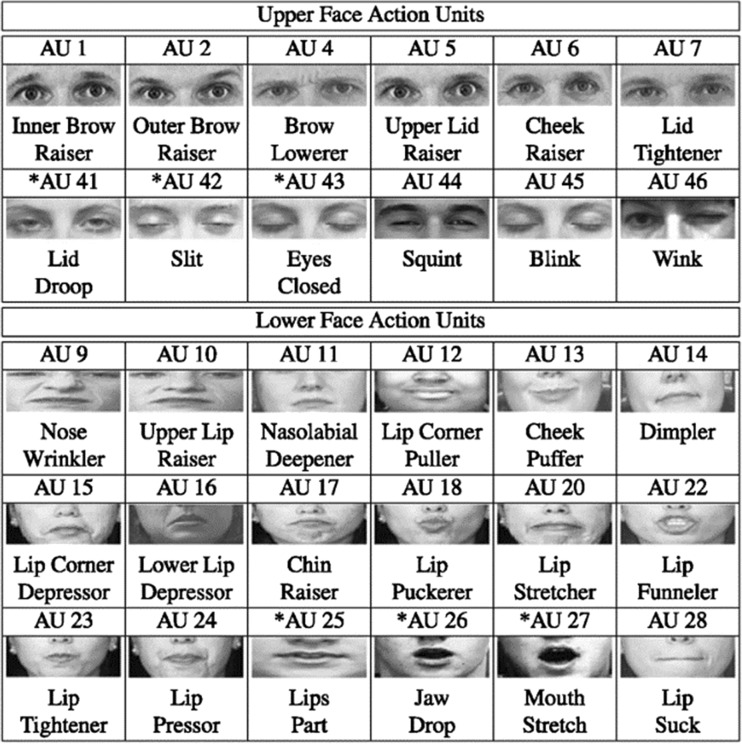
Examples of Actions Units (AU) from Facial Action Coding System (FACS; Ekman & Friesen, 1978) (image from Kanade, Cohn and Tian databases: Kanade, Cohn, & Tian, 2000)

The first studies that used systems to obtain objective measures of facial expression in blind and sighted adults were published in the 1980s. The FACS (Facial Action Coding System) developed by Ekman and Friesen (1978) is a system of anatomical analysis of facial action, in which each movement is deconstructed into Action Units (AU, Fig. 1). All the principal studies conducted since the 1980s have used the FACS or another analogical system of measure like the Maximally Discriminative Facial Movement Coding System (Max; Izard, 1979) to obtain more objective data. Most of these studies used these measurement techniques associated with judgment tasks or recognition tasks by sighted decoders.

The third problem concerns the ecological validity of the task. Galati, Scherer and Ricci-Bitti (1997) highlight important methodological criteria the sighted experimenter should take into account in protocols when using blind participants. Of concern is the choice of scenarios to elicit an emotional response in the specific context of blindness. According to Galati, Scherer and Ricci-Bitti “to say that a person is able or unable to produce the expression of a certain emotion in response to certain stimuli implies that the relationship between that stimulus and that specific emotional response has in some way been established” (Galati, Scherer, & Ricci-Bitti, 1997, p 1367). It is therefore crucial to verify that daily life situations, based on a sighted context, are also valid for blind people.

For instance, Galati and Cattaneo (1995) evaluated whether a sensory deficit, in this case blindness, can affect the perception and evaluation of the antecedent situation, and thus the reaction and emotion it triggers. They asked 19 congenitally blind, 21 visually impaired and 20 sighted adults to recall four episodes in their daily life that caused them to experience an emotion (negative or positive). For each episode, subjects were asked to define the type of emotion felt, its duration and the situation that triggered it. On the whole, sighted and the blind participants reported the same types of emotion (mainly happiness, anger, sadness, and fear). However, differences were found related to the antecedents of these emotions. In the case of fear, sighted subjects reported an equal number of situations in which they themselves were in danger and situations in which someone else was in danger, while blind persons reported only first-person situations. As antecedents of disgust, sighted subjects referred to perceptive contents—mainly visual stimuli—while blind subjects mostly reported situations in which “others behaved in a reprehensible way” (violation of social norms or moral values). They referred to scenarios in which another person did not take into account their disability or refused to provide assistance. A strong link with their disability was also revealed in the antecedents of surprise. In this category, sighted subjects referred to unexpected external events (a visit or news) while blind subjects tended to link surprise emotions with situations in which they accomplished things they did not believe that they would be able to do.

A review of studies of the production of facial expression in blind individuals must take into account these three methodological considerations that may explain some contradictory results. For this reason, we will return frequently to these considerations which are also highlighted in recapitulative tables summarizing the old and recent studies reviewed in this article (Table 1 and Table 2).

### The spontaneous production of facial expressions of emotion from birth to adulthood is independent of visual experience

From the 4th week of life, blind babies smile in response to the sound of their mother's or father's voices (Fraiberg, 1971, 1975, 1977; Freedman, 1964). The smile of blind infants has apparent similarities with the smile of sighted infants, but some differences can be detected concerning its development. At 6 months of age, when the smile of a sighted child becomes relatively automatic in response to the gestalt of familiar human faces, in blind children it is still an irregular smile elicited only by the voice of the mother, or tactile and kinaesthetic stimulation such as tickles and games, for example, bouncing the child on the knee (Fraiberg, 1971, 1975, 1977).

Moreover, authors suggested that despite some similarities, the facial expressive repertoire of blind children is globally limited in comparison to that of sighted children. Between the two opposite emotional states of happiness and anger, there are a wide range of facial expressions marking affect, attention and interest that are differentiated through vision. In a program of intervention in infancy for blind children, Fraiberg (1971) noted different kinds of expressions that are reinforced through visual experience, such as “the expressive look of longing, or the expression that we call ‘quizzical’ or the expression that we call ‘coy’” (Fraiberg, 1971, p. 387).

The absence of these typical signals can be misinterpreted by sighted persons around the child as a lack of interest and affect, sometimes making first parent–child communications difficult. On the other hand, a mother who learns that her baby is blind may also be confronted with psychological pain and this can also be a handicap in the development of the first links of communication. To assist parents who may sometimes be confused by the absence of some typical visual signals, Fraiberg (1975) analyzed very extensively the interactions between blind babies and their surroundings. She showed how their affective behaviors could be very rich if observers will transfer their attention from the face to the child’s body and hands, taking note of the child’s very active manual explorations of familiar faces and objects.

Additional observations were conducted with young blind and deaf-blind children (Eibl-Eibesfeldt, 1973; Goodenough, 1932; Thompson, 1941). Eibl-Eibesfeldt (1973) observed a 5-year-old boy and 7-year-old girl, both born deaf-blind. The same spontaneous expressive behavior was observed, particularly for the expression of happiness. For Eibl-Eibesfeldt, the fact that children with multiple handicaps present the same expressions when they are limited in terms of surrounding visual stimuli, is evidence of the innate nature of this behavior. As we will see, this hypothesis of the universal character of emotions is reinforced by more recent observations of facial expressions elicited spontaneously in blind adults (Matsumoto & Willingham, 2009; Peleg et al. 2006; Tracy & Matsumoto, 2008).

We must note that the majority of the first studies were conducted with a small sample of participants, sometimes only a single case. In children, one of the oldest studies with a representative sample was led by Thompson (1941). By observational and photographic methods, Thompson analyzed facial expressions of happiness, laughing, anger or fear, produced by a group of 26 blind and 29 sighted children, ranging from 7 weeks to 13 years of age. Results showed that even though, in the 1st year of life, blind and sighted children produced very similar facial expressions, a decrease in facial activity was observed in blind children at 2–3 years of age, particularly for smiling and laughing. According to Thompson, neuromuscular patterns of response corresponding to facial expressions appear without the opportunity for visual learning, but social mimicry is apparently responsible for the maintenance of a constant amount of facial activity.

More recently, Tröster and Brambring (1992) used scales of social-development included in the Bielefeld Developmental Test to compare the level of social-emotional development of 22 congenitally blind children and 47 sighted children, aged 9–12 months. Researchers compared the occurrence of different social-emotional behaviors, such as emotions, interactions with the mother, compliance with request, ability to express own needs, etc. Particularly in the area of emotional expressiveness, authors observed that, even if blind children display similar expressions of happiness and anger, their repertoire of expressive reactions is restricted when compared to that of sighted children.

These results are in accordance with Fraiberg’s previous observations (1971, 1975, 1977). Blind children seem to not respond to social stimuli with the same regularity and level of discrimination as their sighted peers. Also, according to mothers of 9-month-old blind children, intensive tactile stimulation often appears to be necessary to elicit pleasure reactions. Differences could also be observed in the child’s reaction to separation from the mother. Compared to sighted children, who frequently protest or cry when the mother leaves the room, these reactions were reported in only 6 of 22 blind children.

More recently, Galati et al. (2001) analyzed spontaneous facial expressions of anger, joy, disgust, surprise, interest, sadness and fear produced by ten sighted children and ten blind children between 6 months and 4 years of age. Blind and sighted children were filmed at nursery school during the course of seven situations selected by researchers and in collaboration with children’s caregivers, who acted in order to generate facial expressions of anger, joy, disgust, surprise, interest, sadness and fear (for instance: “interruption of a behavioral plan”—giving and then removing a biscuit to generate anger; or “contact with a repulsive stimulus”—tasting some drops of lemon juice to generate disgust (Galati et al. 2001, p. 270). A “plurimodal situation” taking into account sensory modalities available in each group of children was also created. Children were surrounded by objects to touch and also objects which produce sounds. Videotapes of facial expressions produced were measured according to a facial muscles measurement system (Max; the Maximally Discriminative Facial Movement Coding System designed by Izard, 1979) associated with a judgment task. Important similarities were found between spontaneous facial expressions elicited in sighted and blind. However, in contrast to results obtained by Thompson in the 1940s, Galati, et al. (2001) did not find a decrease with age in the facial expressiveness of blind chil-dren. In judgment tasks, decoders globally attributed the same correct emotional label to facial expressions produced by both groups of subjects. Few differences were found, with the ex-ception of sadness and fear, which were systematically con-fused with one another in expressions elicited in blind chil-dren. Interpretations of these emotions have been more diffi-cult because they appear later in emotional development (between 6 and 12 months of age for sighted), and this delay is probably even greater in children who are blind (Galati et al. 2001). An additional interesting explanation for this confusion may be “a sadness sensation felt by judges themselves in recognizing the faces of the blind children” (Galati et al. 2001, p. 275). This tendency to attribute sadness to blind children’s expressions could have influenced judges in applying this label for other expressions.

**Fig. 2:**
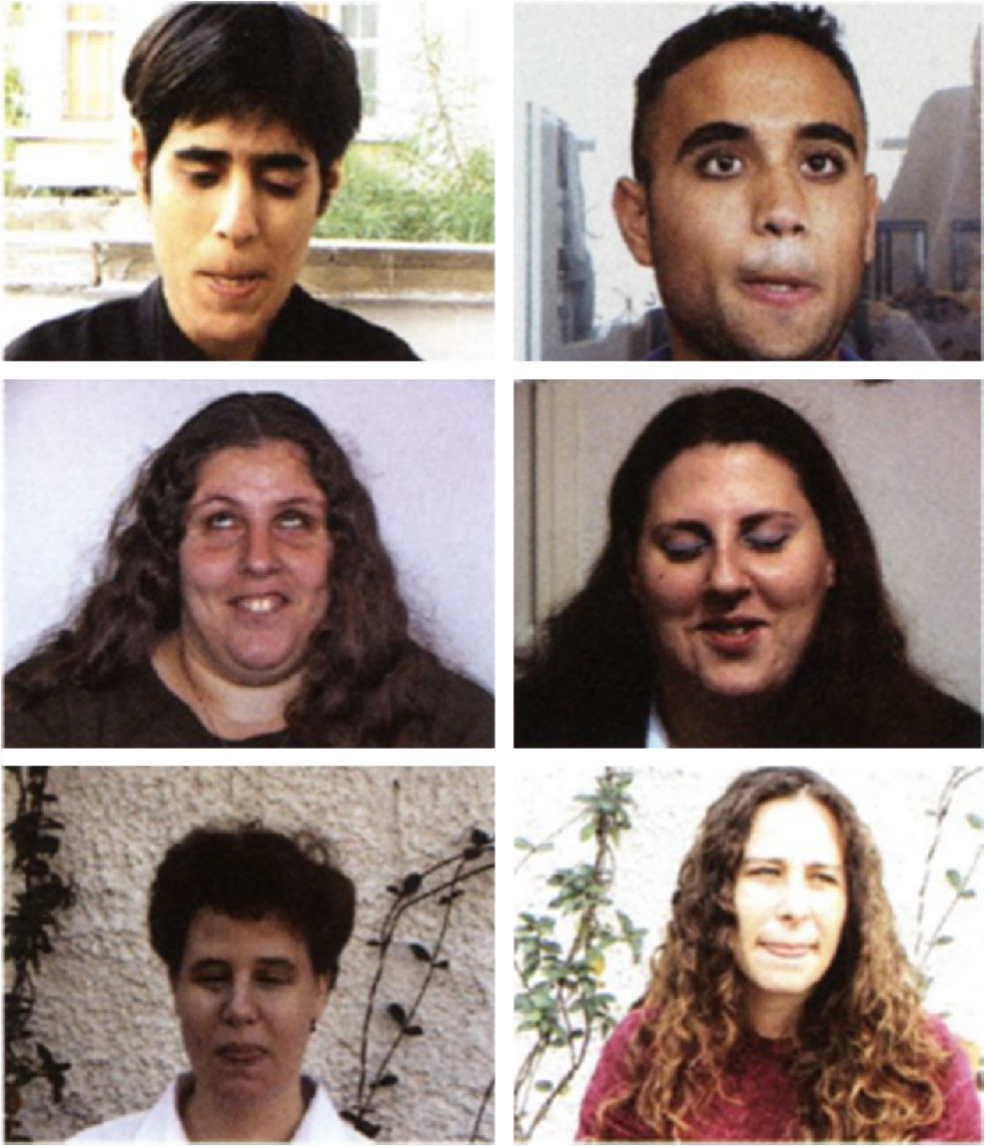
Similar movements in born-blind participants (*Left*) and their sighted relatives (*Right*) documented by Peleg et al. (2006). Copyright (2006) National Academy of Sciences U.S.A﻿.

In adults, Peleg et al. (2006) examined the facial configurations of 21 congenitally blind individuals and their sighted family members or sighted strangers (31 sighted participants in total). Using two types of analysis (documentary and computational), results revealed correlations between the facial configurations of congenitally blind (who have not touched their relatives' faces, during any stage of their life, in order to adopt facial expressions) and sighted people. Moreover, results revealed more correlations between the facial configurations when individuals belonged to the same family, particularly for think-concentrate, sadness, and angry facial expressions (Fig. 2). These data provided evidence for a “unique family facial expression signature” and evidence for a hereditary basis for facial expression (Peleg et al. 2006).

Tracy and Matsumoto (2008) tested whether sighted, blind and congenitally blind individuals across cultures spontaneously display pride and shame behaviors in response to the same success and failure situations—victory and defeat at the Olympic or Paralympics Games. Results showed that sighted, blind, and congenitally blind individuals from 37 nations displayed the behaviors associated with a prototypical expression of pride in response to success. Sighted, blind and congenitally blind individuals from most cultures also displayed behaviors associated with shame in response to failure.

**Fig. 3:**
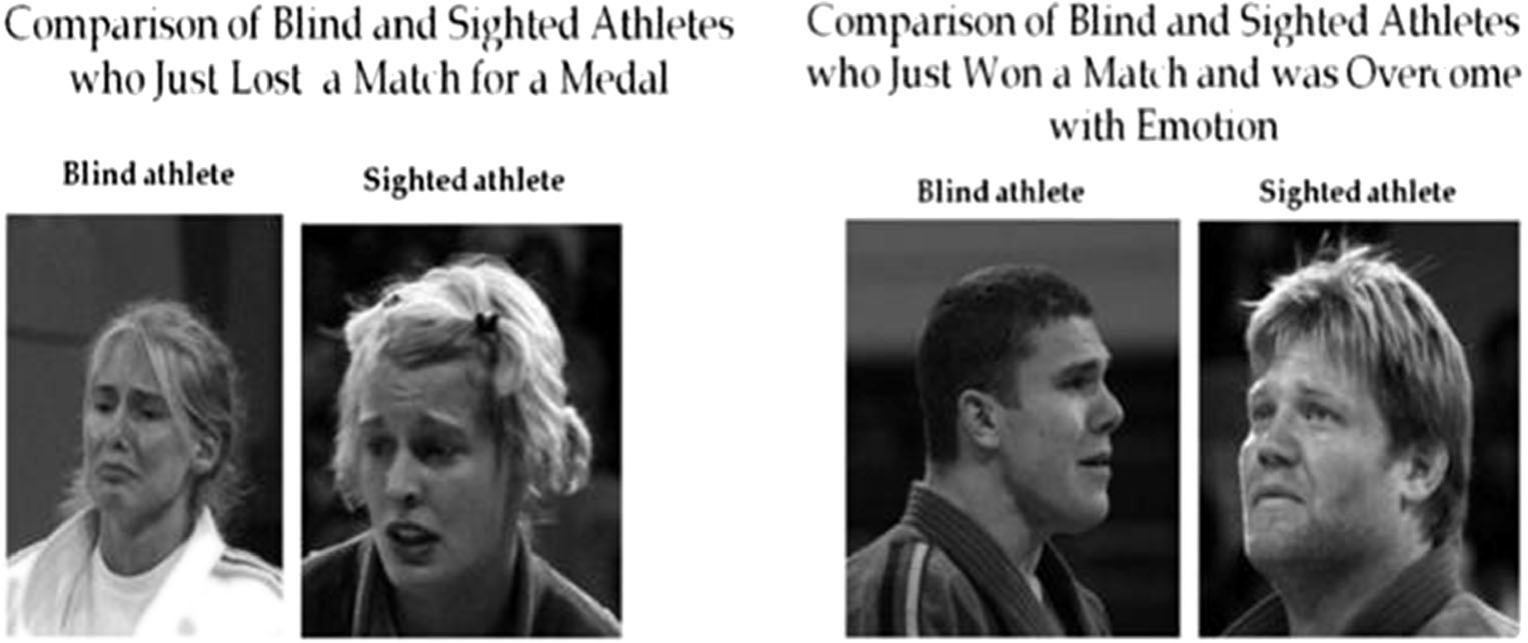
Comparison of facial expressions produced by blind and sighted athletes (Matsumoto & Willingham, 2009). Reprinted with permission from Bob Willingham.﻿

In another study, Matsumoto and Willingham (2009) examined facial expressions posed spontaneously by congenitally and late blind judo athletes in the Athens Paralympics Games, comparing them with expressions posed by sighted athletes in the same game context. Facial expressions of blind athletes from 23 cultures were photographed during three different evocative emotional situations in the championship: at the end of the match and at two moments during the medal ceremonies (Fig. 3). These situations involve different emotional responses considering their social nature. For example, expressions of sadness or disgust can be expected in athletes who lost at the end of the gold medal match. These same athletes (silver medalists) will nonetheless probably display non-Duchene smiles (a social smile) when receiving the silver medal. Photographs were coded with FACS, and the results of this ecologic and very pragmatic study revealed that blind athletes produced the same facial configuration to show emotion as did sighted athletes. There were also no significant differences found between congenitally and late blind groups. The only differences found were related to head movements in blind individuals, which according to the authors were associated with an attempt to maximize audio stimuli, and other typical behaviors like eye rotations or eyebrow raises. As mentioned earlier, these can produce some uncommon static features in photographs signaling differences of facial musculatures in FACS coding.

The fact that blind athletes used smiles and facial control in social situations in this study in the same ways that sighted athletes did suggests that observation is not necessary for individuals to learn how to regulate their expressions. Some studies observed whether this ability to spontaneously mask an emotion also occurs in infancy. In a study from Cole, Jenkins and Shoot (1989), 12 congenitally blind children and 12 sighted children, aged 6–13 years, were instructed to choose their preferred toy from a list. Experimenters examined videotapes of the expressions posed by children who received toys they did not want as well as their verbalizations about expressive control over their disappointment. To examine verbalizations, the child was interviewed about his feeling (“How did you feel when you got that prize?”) and about the knowledge of another person, present during the situation, concerning the child’s feeling (“Did she know how you felt?”, “How did she know or not know your feelings?”). Results showed that blind and sighted children have the same capacities to mask a negative emotion with a false smile that has been observed in adults (Matsumoto & Willingham, 2009), but blind children were less likely to refer spontaneously to control their facial expression. Seven (58.3%) of the sighted children referred to facial control; in contrast, one (8.3%) blind child made this reference. Blind children referred to verbal control of their disappointment (e.g., “I didn't tell her”) rather than facial expressive control.

In the same manner, Galati et al. (2003) compared spontaneous emotional facial expressions of blind and sighted children between 8 and 10 years of age to verify their capacity to modulate and control facial behaviors in complex emotional situations. Children were filmed in their classroom in the midst of seven situations eliciting emotions. These situations were the same as those selected in the study presented earlier involving younger children (Galati et al. 2001) with only some adaptations with respect to age (for example, “interruption of a behavioral plan” the situation of removing a biscuit, was replaced by the interruption of a game). Facial expressions were coded using FACS. Results showed that despite the fact facial expressions of blind and sighted children were very similar, some differences emerged. Firstly, and in general, the total frequency of activated AUs was higher for blind than for sighted children (314 for the blind, 253 for the sighted). Secondly, sighted children masked negative emotions more frequently than blind children. The activation of the dimpler (AU14/mouth corners, see Fig. 1), is a socially shared code used to control and/or mask anger in facial expression (Ekman & Friesen, 1978) and is more frequently observed in sighted children than in blind children. Finally, FACS coding showed some specific AUs that prevail in blind children’s faces, such as head movements, eye closure and mouth opening associated with “blindisms”. According to the authors, these results suggest that even though similarities were found between expressions produced by blind and sighted children, blind children conform less to the display rules that determine in which situations, which emotions should be expressed or masked and the degree of control exercised over their intensity.

In summary, old and recent studies revealed that blind and sighted people spontaneously produce the same type of facial expression, particularly for basic emotions like happiness, sadness and fear. Furthermore, some differences were found concerning the occurrence of these expressions in infancy. Concerning the capacity of blind subjects to conform to display rules, such as masking a negative emotion with a positive one, a study of adults did not find differences associated with visual experience (Matsumoto & Willingham, 2009). This capacity was also observed in blind children, even though they mask negative emotions less frequently than sighted children (Galati et al. 2003), and verbalize less about this expressive control (Cole et al. 1989).

However, as we will see in the next section, whereas visual experience seems unnecessary to spontaneously produce adequate facial expressions, it does seem to affect the production of voluntary expressions.

### The voluntary production of emotional facial expressions are affected by visual experience

In a study comparing spontaneous and voluntary expressions produced by blind individuals, Dumas (1932) suggested that expressions elicited by a real emotional sensation appear to be associated with innate cognitive programs while voluntary expressions are connected with prior visual observation. Dumas’ ideas are then reinforced by Fulcher (1942) in a study of 50 congenitally blind and 118 sighted individuals, males and females, aged 4–21 years. In terms of a developmental view, results showed that the voluntary expressions of blind infants are less pronounced but are still similar to those of sighted but become inadequate at adulthood. Even if blind and sighted subjects use the same facial movements to reproduce emotions, in the first group these movements are less clear and their muscles less pronounced. Webb (1977) obtained similar results in a study aiming to train blind individuals to execute facial expressions. Before training, voluntary expressions produced by subjects received poor rates of recognition by judges. Data revealed a performance improvement following training with a device that allowed participants to have audio feedback while they produced facial movements and made efforts to control them.

In research comparing voluntary and spontaneous expressions generated by seven congenitally blind children and seven sighted children, aged 6–13 years, Ortega, et al. (1983) showed that blind and sighted spontaneously produce the same action units for the smile. The action unit 12 (lip corner puller, see Fig. 1) was present in 97% of photographs of smiles produced by blind participants and in 96.2% of those produced by sighted participants. The AU 6 was also present but less frequently (46.7% for blind and 79.5% for sighted).

Concerning voluntary expressions, Ortega et al. (1983) asked another group of 15 blind and sighted subjects to produce six facial expressions (surprise, happiness, sadness, anger, fear and disgust). The photographs were evaluated by 44 judges and also measured with FACS. The highest percentage of recognition by judges of expressions produced by blind participants was about 40% (46% for sadness and 40% for happiness). Expressions of surprise and fear obtained very low rates of recognition (11% and 15%, respectively). In expressions produced by sighted, percentages of recognition were globally higher (82% for disgust, 68% for happiness, 38% for fear). Moreover, measures from FACS revealed that facial muscles of sighted matched more clearly with the expected prototypical patterns of basic emotions postulated by Ekman and Friesen (1978) for the expressions of surprise, happiness, anger, fear, and disgust.

Results obtained by Ortega and collaborators seem to confirm a dichotomy between spontaneous and voluntary expressions as revealed in previous studies. However, according to the authors, these results should be interpreted with caution. They noted that the differences between blind and sighted individuals are quantitative and not qualitative. Some expressions produced by blind participants, like happiness and sadness, are better recognized by judges than others, and, particularly for sadness, the percentage of recognition is almost identical to the percentage rate for sighted participants. As in Galati et al. (2001), a common denominator of “unhappiness” associated by judges with the facial expressions of blind individuals can explain these results.

In a study examining the production of voluntary basic emotions by 20 blind and 20 sighted children, aged 4–12 years, Roch-Levecq (2006) observed that, even though blind children were just as able as sighted children to understand the underlying cause-effect relationships that evoke basic emotions, their expressions did not convey these basic emotions to others as well as those of sighted children. Indeed, sighted children were more able to convey fear and happiness than the blind children, and results suggested that it was significantly more difficult for the adult raters to discriminate the blind children's facial expressions, even to differentiate between positive and negative emotions, compared with the facial expressions of sighted children. However, as noted in the methodological considerations, Roch-Levecq used a visual judgment task and results stemming from these tasks must be interpreted with caution.

In adults, Rinn (1991) videotaped facial expressions posed by 20 congenitally blind and 20 sighted subjects at the request of the experimenter (voluntary condition) and in a structured interview consisting of asking participants to interpret 20 old sayings (spontaneous condition). The videotapes were analyzed with FACS. Results showed that the facial expressions of congenitally blind are of generally poor quality when they voluntarily pose facial expressions, whereas their expressions produced during the interview presented facial movements similar to sighted participants (particularly smiles and eyebrows). To explain these results, Rinn proposed a hypothesis of two distinct areas of the brain. He suggested that spontaneous expressions depended on innate processes originating from the subcortical areas while voluntary facial expressions were connected with control-specific processes emanating from the cortical motor strip. This voluntary control seems to depend on visual feedback regarding the position of facial landmarks. This neuropsychological approach was also assumed in a recent review (Hwang & Matsumoto, 2016).

Still in adults, Galati, Scherer and Ricci-Bitti (1997) compared the ability of 14 congenitally blind and 14 sighted adults aged 20–70 years to voluntarily produce facial expressions related to a number of emotions using both objective facial measurement and observer recognition. Scenarios to elicit an emotional response in the specific context of blindness were chosen based on data obtained in previous studies we mentioned above (Galati & Cattaneo, 1995). However, it is unfortunate that the list of scenarios selected from these previous works is not presented in the study. Given this, it is difficult to evaluate if the ecologic validity of the stimuli has been effectively implemented and its impact on performances. FACS measurements revealed less difference than one might have expected on the basis of previous studies between blind and sighted persons in voluntary facial expression. However, as in previous studies, expressions of blind individuals were poorly recognized by judges. Once again, a judgment problem cannot be excluded. According to Galati, Scherer and Ricci-Bitti, sighted judges may have been confused by unusual habitual expressions of many of the blind individuals, that is, the same differences founded by Matsumoto and Willingham (2009) in their analysis of spontaneous facial expressions of blind individuals.

The effect of “blindisms” on the expressiveness of blind persons was further detailed in a recent study examining spontaneous play interactions between seven visually impaired mothers and their sighted children, aged between 6 months and 3 years (Chiesa et al. 2015). The interaction was filmed with two cameras, one for each partner’s face and upper body. Using a control group of sighted mothers and their sighted children, four modalities of communication were compared: voices, facial expressions, physical contacts and gaze. Analysis of facial expressions using the FACS showed differences between sighted and visually impaired mothers only with respect to forehead movements. Mothers with visual impairments produced very irregular movements of eyebrow raises and frowns, much less or much more often than sighted mothers. Another quantitative analysis described in more detail the meaning of eyebrow movements produced by a congenitally blind mother compared with a sighted mother. Based on categories of facial events suggested by Ekman and Friesen (1978), results showed that 61 of 72 of the eyebrow movements of the blind mother were mostly stereotypical repetitive movements not linked with emotional or communicational meaning.

**Fig. 4:**
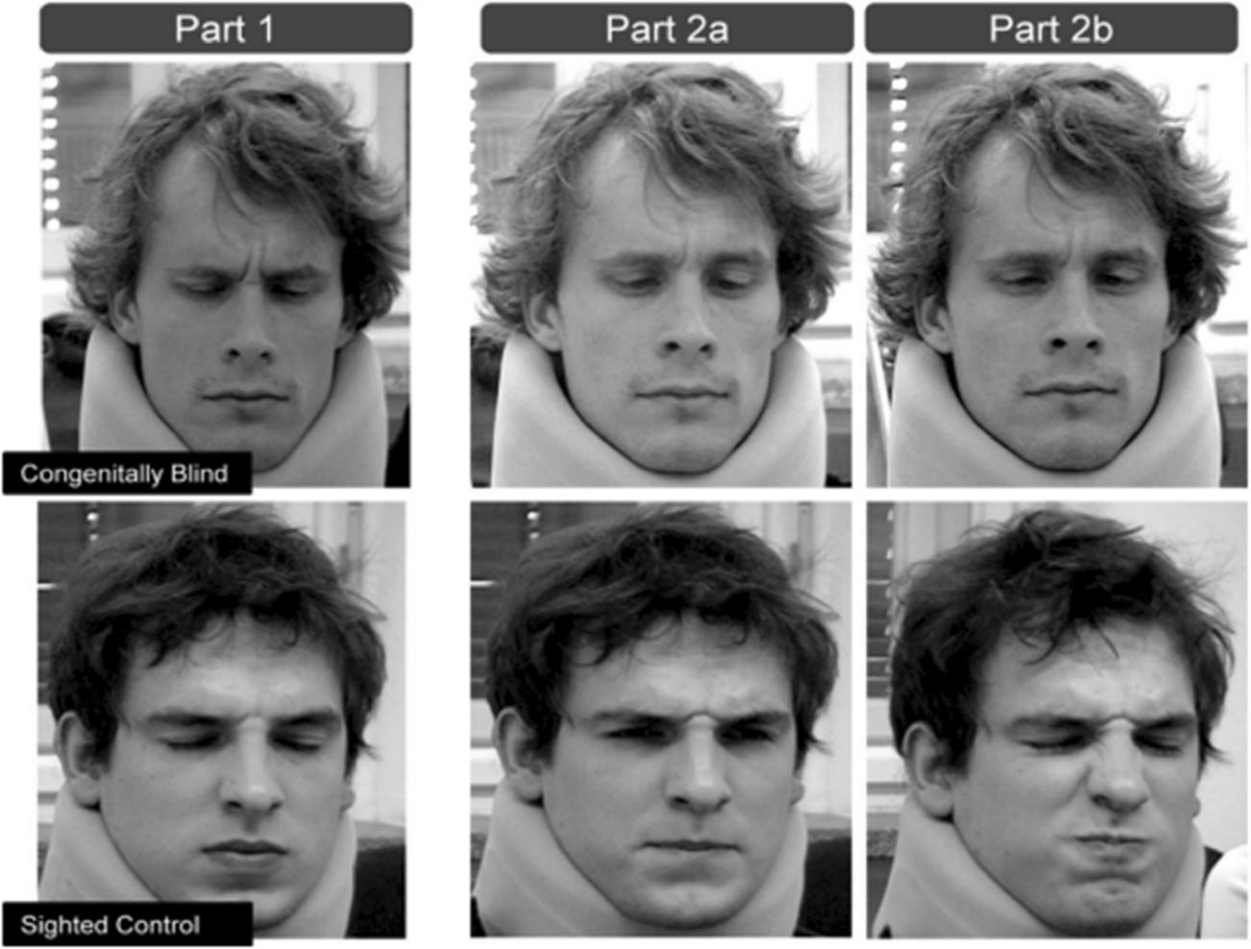
Examples of evoked (part 1) and voluntary facial expressions (part 2a: reproduction, part 2b: optimal condition) produced by a blind and a sighted subject (image from Kunz et al. 2012). Copyright (2012), with permission from Elsevier

In another study comparing evoked and voluntary facial expressions of pain produced by 21 blind and 42 sighted adults, Kunz and colleagues (2012) highlighted the impact of display rules on the expressiveness of blind persons. In the first experimental condition, subjects received thermal stimulations in three intensities: close-to-painful, slightly painful or moderately painful (Fig. 4). The term “evoked expressions” is preferred to “spontaneous expressions”, in this case to evoke a condition in which a real emotion is elicited through stimulation in a laboratory. In the second condition, aimed at analyzing voluntary expressions, subjects were asked to reproduce the facial expression elicited in part 1 of the study (2a, reproduction), and to voluntarily express as optimally as possible what they had been feeling during part 1 of the study (2b, optimal condition).

Results showed that, in an evoked condition, congenitally blind and sighted individuals displayed the same pattern of facial movements in response to painful stimulation, but blind subjects were facially more expressive compared to sighted. In contrast, they showed rather reduced voluntary facial expressions. Blind individuals were not able to voluntarily increase their facial expression when asked to pose an “optimal expression” as sighted will do. Interesting explanations related to display rules for the expression of pain in the culture were provided. Differences in intensity that were found seem to be related to the capacity to simulate and control these behaviors in infancy. Children are, in general, discouraged from expressing emotions with great intensity, and they also learn to mask pained expressions to avoid embarrassment in front of peers. In contrast, they also learn to voluntarily exaggerate their facial expression of pain in some situations. This capacity to regulate the intensity of painful facial expressions, raising or lowering the level of expression, seems to be linked with visual—and social—feedbacks.

In summary, studies about voluntary production revealed that blind individuals have difficulty posing emotional expressions. However, according to some authors, these results must be interpreted with caution. Difficulty in posing emotional expressions seem apparent for some facial expressions and not others (Ortega et al. 1983). The lack of ecologic validity of the stimuli (Galati, Scherer, & Ricci-Bitti, 1997) and the influence of facial behaviors specific to blindness, known as “blindisms”, in the interpretation of facial expressiveness may partially explain the poor performances of blind subjects (Chiesa et al. 2015; Galati, Scherer, & Ricci-Bitti, 1997; Matsumoto & Willingham, 2009; Ortega et al. 1983).

## General discussion and perspectives

This paper reviews twenty-one older as well as recent studies that have examined the production of facial expressions by blind individuals. Of these, fifteen reported that blind subjects spontaneously produced the same types of emotional expressions as sighted individuals, particularly for basic emotions like happiness, sadness and fear (Chiesa et al. 2015; Cole et al. 1989; Dumas, 1932; Eibl-Eibesfeldt, 1973; Fraiberg, 1975, 1977; Freedman, 1964; Galati et al. 2001; Galati et al. 2003; Goodenough, 1932; Matsumoto & Willingham, 2009; Ortega et al. 1983; Peleg et al. 2006; Thompson, 1941; Tracy & Matsumoto, 2008; Tröster & Brambring, 1992). This similarity in expressiveness is also evidenced in a more recent study on facial expressions of pain (Kunz et al. 2012). In infancy and childhood, however, some differences were found concerning the occurrence of these expressions. The repertoires of expressive reactions are more restricted in blind children in comparison with sighted children (Fraiberg, 1975, 1977; Tröster & Brambring, 1992). A decrease in facial activity in blind children detected in early observations (Thompson, 1941) was not confirmed in more recent work (Galati, Miceli, & Sini, 2001).

Among these twenty-one studies, eight examined voluntary expressions and indicated that blind individuals have difficulty posing emotional expressions and controlling their intensity (Dumas, 1932; Fulcher, 1942; Galati, Scherer, & Ricci-Bitti, 1997; Kunz et al. 2012; Ortega et al. 1983; Rinn, 1991; Roch-Levecq, 2006; Webb, 1977). However, this difficulty is apparent for some facial expressions and not others. Attention paid to ecologic validity of the stimuli seems also to have influenced the performances. Moreover, some problems regarding judgment of the expressions of blind individuals by sighted decoders were also advanced by some researchers (Galati, Miceli, & Sini, 2001; Ortega et al. 1983). Actually, sighted judges could be confused by the occurrence of “blindisms” reflecting unusual and habitual facial movements of many blind individuals and/or be influenced by a feeling of “unhappiness,” experienced by the judges themselves, as they viewed the expressions of blind individuals. Differences noted between facial expressions produced by blind and sighted individuals, based on these subjective judgments, were not confirmed by more objective systems of measurements of facial configurations (Galati, Scherer, & Ricci-Bitti, 1997; Matsumoto & Willingham, 2009).

Globally, at least with respect to facial patterns elicited by blind persons in real emotional contexts, the studies reviewed provide compelling evidence for a non-visually learned and universal source for facial expressions. However, we think that three questions should be explored in future research to reinforce this point of view. The first question concerns the link between production and recognition of facial expressions in blind individuals. In light of the grounded cognition approach, it could be interesting to examine whether the production of expressions can have an impact on their ability to discriminate facial expressions by touch. Regarding the link between perception and production, we can ask if other sensory channels and modalities of learning, such as touch and hearing, might explain the production of adequate facial expressions in the absence of vision. The second question concerns the role of display rules in this context. Some researchers showed that even if blind subjects produce the same facial patterns as sighted ones, visual feedback could still be important in shaping this innate expressions. Finally, we also discuss the role of other channels to express emotions in the absence of visual signs.

### The link between production and recognition of emotions by other sensorial channels

Studies reviewed offer compelling evidence that visual learning seems to not be necessary in order to produce the same pattern of facial expressions in real emotional contexts. However, results did not answer the question of whether the processes implemented in blind people are the same as those implemented in sighted people. Particularly, one may wonder if other nonvisual processes, including vocalizations and tactile cues, might play a role in the context of blindness, not only in producing but also in discriminating the facial expressions of others.

The link between production and perception is already well established in the studies on emotional expression in sighted (for a review, see Niedenthal, 2007). This link is particularly highlighted in studies about facial mimicry of smiles (Korb et al. 2015; Niedenthal, Mermillod, Maringer, & Hess, 2010). When we observe a facial expression, we often recreate the motor production of the perceived facial expression in our own faces. In light of the grounded cognition approach (Barsalou, 2010), the hypothesis advanced is that the experience of performing facial expressions contributes to the perceptual process and the meaning assigned to the expressions perceived. A recent study also evaluated top-down social influences on the embodied processing of facial expressions. Results showed that facial mimicry is reduced in the absence of social utility (Beffara et al. 2012).

It would, therefore, be interesting to know if this link between production and perception can also take place in the context of blindness through other sensory channels, such as touch or hearing and the role of top-down and bottom-up processes in this context. Does the ability to produce adequate facial expressions have an impact on the ability of blind individuals to discriminate expressive faces by touching the other’s face or by hearing emotional tones of voice? Or, examining the question in another sense, does the opportunity to perceive facial expressions through other sensorial channels have some impact on the ability of blind individuals to produce correct emotional feedback?

Actually, only a few studies have been conducted to explore the discrimination of emotional faces by touch and the question remains open. For example, studies examining the capacity of touch to discriminate expressive faces (Kilgour & Lederman, 2002; Lederman et al. 2007; Lederman et al. 2008; Picard, Jouffrais, & Lebaz, 2011) provided evidence that touch performs better than would be expected as a means of discriminating facial expressions as displayed in real faces, tactile facemasks (Kilgour & Lederman, 2002; Lederman et al. 2007) and even in tactile drawings of facial expressions (Lederman et al. 2008; Picard et al. 2011). It is important to note that the main focus of studies concerning discrimination of faces by touch has been to examine perceptual mechanisms of the haptic sense and its differences and/or similarities to vision. The impact of embodied cognition in the recognition of facial expressions across different senses is not the central question here. However, the very good performance of touch in these tasks, particularly when compared with the very low discrimination rates obtained in other tasks using drawing of common objects (Lederman, Klatzky, Chataway, & Summers, 1990) may indicate an effect of the production of facial expressions in their own faces in the performances of the blind. It is an interesting direction to explore in future research about the production and perception of emotions in blind and sighted subjects.

Concerning the influence of tactile perception of faces in the production of adequate facial expressions, the current state of research does not provide a clear answer. Results of studies mentioned about tactile recognition of emotional faces provided evidence on how touch performs per se in perceptive tasks, but we know less about the way and with what regularity blind individuals use this sensorial channel to obtain information about the emotional states of others. Moreover, except in Picard et al. (2011), all studies were conducted only with blindfolded sighted subjects. Even if touch seems to be very important in forming the first links between blind babies and their parents (Fraiberg, 1977), in our culture characterized by some prudishness and taboos about touching (it is not advisable to touch persons whom we do not know intimately), we can easily suppose that blind persons do not touch the expressive faces of others in each and every social interaction. In this direction, intervention programs with tactile educational tools allowing one to touch emotional faces may allow improvement of expression of emotional faces by blind people.

Another very interesting topic, not sufficiently explored, relates to the role of sounds and tones of voice used by blind individuals in understanding the emotional states of others. Minter, Hobson and Pring (1991) tested the ability of eight congenitally blind children (aged 6–12 years) to identify vocal expressions of emotions. Unexpectedly, results showed that in comparison to sighted children, blind children had difficulty recognizing emotions related to vocal sounds. Similar results were obtained by Blau (1964) and Dyck, et al. (2004) in tasks assessing the ability of blind children and adolescents to recognize and to understand emotion. While they have very good scores on tasks that depend on verbal comprehension of emotions, as elicited in different situations and dialogues, they have difficulty recognizing emotional expressions based on hearing tones of voice.

How is it possible to explain the poor performance of blind individuals in identifying emotion through another’s tone of voice? Considering that this identification depends directly on their intact sense, the cause of this deficit is not clear. The ability of blind children to recognize emotion is less than would be expected on the basis of their good performance in verbal tasks indicating emotional understanding and their use of emotional vocabulary (Dyck et al. 2004). Therefore, blind children were less able to identify the sounds of emotions than they were to identify sounds of “nonemotional” objects (i.e., vehicles or animals, Minter, Hobson, & Pring, 1991). A hypothesis advanced by Dyck, et al. (2004), and shared by Peterson, et al. (2000) and Minter, Hobson and Pring (1991), is that blindness could affect gaining access to important cues during conversations about emotional states. This lack of access resulted in delayed acquisition of a theory of mind (Dyck, et al. 2004). In sighted children, facial expressions, gestures and vocalisations are perceived in an integrated package that allows them to make connections between different facets of emotions and how emotions are related to behaviors (Minter, Hobson, & Pring, 1991). The role of context in the perception of emotion was highlighted in a recent study about facial emotion recognition by sighted children (Theurel et al. 2016). Thus, blind children who have limited input in this regard may have more difficulty in differentiating emotional tones of voice when these are presented in an isolated manner.

To conclude, while studies with blind individuals offer compelling evidence for a non-visually learned and universal source for facial expressions, more research is clearly needed to determine whether other specific processes could be influencing the ability of these subjects to express facial emotions in the same way as sighted individuals. Even if we can argue that the impact of other learning mechanisms is limited when compared with the opportunity sighted individuals have to see expressive faces and imitate emotional expressions observed in others (Eibl-Eibesfeldt, 1973; Galati, Scherer, & Ricci-Bitti, 1997; Hwang & Matsumoto, 2016), the role of sensory channels like touch or emotional vocalizations cannot be completely excluded on the basis of the present review. Future research can supply new strong evidence to the debate regarding the innate or the culture-constant learning character of the production of emotional facial expressions by blind individuals.

### Variability concerning display rules in facial expressiveness of blind people

Some studies providing evidence that blind individuals expressed the same pattern of facial expressions as sighted individuals also highlighted some variations that reflect the context of blindness and differences in the intensity and control of emotions in some specific situations. Blind individuals seem to conform less to the display rules linked with visual feedbacks to determine in which situations, which emotions can be expressed and control over their intensity (Galati et al. 2003; Kunz et al. 2012). This variability within the same facial pattern is consistent with the concept of display rules proposed by Ekman (1993). Display rules are dictated by norms and social and cultural pressures that can affect facial expressiveness in different ways: individuals can modulate their facial expression by extenuating, neutralizing or masking it (Tcherkassof, 2008).

Interesting results were provided by Kunz et al. (2012) concerning variations in the intensity of expressions of pain by blind persons. Data obtained regarding facial expressions of pain by blind adults provided evidence that visual learning seems to be a prerequisite in order to up-regulate and also to down-regulate these expressions. Future research studies were requested to examine if variations in intensity due to visual feedback are also present in basic emotions such as fear, happiness or sadness, and to what extent.

More studies were also requested to examine the capacity to mask a negative emotion in the context of blindness. Only three studies reviewed were devoted to this question. While one study suggested that blind adults have the same capacities as sighted subjects to mask an emotion in a social context (Matsumoto & Willingham, 2009), studies with children provided evidence that they mask negative emotions less frequently than sighted children (Galati et al. 2003) and verbalize less about this expressive control (Cole et al. 1989). It could be interesting to dig deeper into this question by evaluating, for example, how this expressive control ability develops in blind individuals from birth to adulthood and to what extent visual feedback or other learning processes, such as verbal feedback, takes place in this case.

The impact of display rules in the expressiveness of blind subjects reflects also the occurrence of “blindisms” (Brambring & Tröster, 1992; Leonhardt, 1990). Some studies pointed out that these stereotypic and repetitive behaviors can produce some facial features that might be misinterpreted by a sighted interlocutor (Chiesa et al. 2015; Galati, Scherer, & Ricci-Bitti, 1997). Future research is also invited to expand upon this point and to detail the impact of these behaviors on the communication of emotional states by blind people.

### Use of other channels to express emotions in the context of blindness

In a Galati and Cattaneo (1995) study about the antecedents of emotions in the context of blindness, another interesting difference was revealed when subjects reported their reaction when confronted with an emotion. It has been shown that sighted subjects talk more often about behavioral reactions (expressions and postures) while blind subjects refer mostly to verbal and cognitive reactions (Galati & Cattaneo, 1995, p 41). These results suggest that blind persons can envisage differently the functional and expressive character of emotional feelings and use signs other than facial expressions to express their emotions, such as tone of voice, verbal behaviors or physical contacts.

In their study about communicative interactions between visually impaired mothers and their sighted children, Chiesa, Galati and Schmidt (2015) showed that they use compensatory strategies to guarantee a harmonic interaction. Indeed, physical contact and verbal productions assume an important role in this context. Moreover, sighted children are able to adapt their modalities to communicate when they are in interaction with their visually impaired mothers. They direct their gaze less frequently to their mothers than to sighted interaction partners, thus adapting differential modes of communication. These data are consistent with the results of extensive observations made by Fraiberg (1971, 1975, 1977) of the interactions between blind babies and their mothers or caregivers. According to Fraiberg, a rich vocabulary of non-visual signs is triggered between blind babies and adults to ensure good communication and the development of emotional attachment. An important component of this vocabulary is body language. She gives several examples of how hand behaviors are signs of affection and interest in blind babies. New research studies are requested to examine in more detail these differential modes of emotional expression, beyond what is apparent only in the face and visage of the participants, when the context is one of blindness.
